# An Investigation Into the Effects of Destination Sensory Experiences at Visitors’ Digital Engagement: Empirical Evidence From Sanya, China

**DOI:** 10.3389/fpsyg.2022.942078

**Published:** 2022-07-05

**Authors:** Jin Ai, Ling Yan, Yubei Hu, Yue Liu

**Affiliations:** ^1^School of Business Administration, Faculty of Business Administration, Southwestern University of Finance and Economics, Chengdu, China; ^2^School of Public Administration, Southwestern University of Finance and Economics, Chengdu, China; ^3^Warwick Manufacturing Group, University of Warwick, Coventry, United Kingdom; ^4^Business School, Sichuan University, Chengdu, China

**Keywords:** sensory experience, digital engagement, place attachment, place dependence, place identity

## Abstract

This study investigates the mechanism of how sensory experiences influence visitors’ digital engagement with a destination through establishing a strong bond and identification between a destination and tourist utilizing a two-step process. First, visitors’ sensory experiences in a destination are identified through a content analysis of online review comments posted by visitors. Afterward, the effects of those sensory experiences on visitors’ digital engagement through destination dependence and identification with that destination are examined. Findings suggest that sensory experiences are critical antecedents of visitors’ bond and identification with a destination. Visitors’ positive destination-related sensory experiences increase their dependence on and identification with the destination, and this dependence and identification positively influence their digital engagement behavior on social media.

## Introduction

Customer engagement refers to long-term and ongoing close interactions between customers and product/service providers (Zhou et al., 2020) that reflects customers’ emotional, psychological, and behavioral connection to a brand (Brodie et al., 2011; Dessart et al., 2015). It plays critical roles at each stage of the customer life cycle such as acquiring, converting, retaining, and turning customers into advocates for a brand. Furthermore, customer engagement has significant effects on consumer outcomes such as involvement (Hollebeek et al., 2014), satisfaction (Bowden, 2009; Van Doorn et al., 2010), loyalty (Thakur, 2016; Harrigan et al., 2017), and word-of-mouth (WOM) behavior (Matos and Rossi, 2008).

Since the level of engagement is heavily dependent on successfully building mutually beneficial and engaging relationships with customers in an appropriate, effective, and meaningful way, brands are increasingly utilizing digital social media (e.g., Facebook, Twitter, and Instagram) as the number of customers who use those social media sites keeps increasing (Kim and Kim, 2019). As a result, brands, in recent years, have been committing significant resources to social media activities that can help them improve customers’ “digital engagement” with their brand (Meire et al., 2019).

Like other brands, visitors’ positive emotional and behavioral engagement with a destination is critical since it can promote destination experience, trust and loyalty (So et al., 2012; Barnes et al., 2014; Ahn and Back, 2018; Villamediana-Pedrosa et al., 2020), emotional commitment (Lee et al., 2016; Han and Hyun, 2017), satisfaction (Mistilis and Gretzel, 2013; Cabiddu et al., 2014), and positive WOM behavior (Filieri et al., 2015; Hudson et al., 2015), and further contribute to the success and prosperity of destinations. Therefore, visitor engagement, especially visitors’ digital engagement, has become an important topic for tourism researchers and practitioners since a large portion of travelers read the comments posted on review sites and experiences shared on social media before making their purchase decisions.

Research suggests that customers’ level of engagement with a brand is often influenced by customers’ experience (So et al., 2012; Ahn and Back, 2018). Thus, visitor experience, especially the sensory experience, has been attracting increasing attention due to their impact on visitors’ attitudes and behaviors (Agapito et al., 2014). Sensory experience refers to visitors’ overall perceptions of goods or services that are experienced collectively through visitors’ five senses (vision, auditory, olfaction, taste and tactile) (Krishna, 2012; Lv et al., 2020a). These sensory experiences are critical components of overall experiences that can have a significant impact on visitors’ satisfaction in addition to non-sensory experiences, such as affective experiences, behavioral experiences, and intellectual experiences (Barnes et al., 2014).

Visitors form their overall sensory experience perceptions of a destination through participating in activities that help them learn and develop a greater understanding and appreciation of a destination by using their five senses, i.e., the visual, auditory, taste, olfactory and tactile stimuli of the destination. The sensory experience created collectively by these sensory elements influences visitors’ cognitions and, subsequently, shapes their identification and attachment to a destination (Agapito et al., 2014; Lv and Wu, 2021). Since these five senses play an significant role in human lives, delivering tourism experiences that satisfy all five senses would assist destinations in establishing strong sensory relationships with visitors (Lv et al., 2020a).

Delivering positive sensory experiences can further reinforce visitors’ positive feeling and cognition toward a destination, and ultimately yield positive psychological, and behavioral responses toward the destination (Krishna, 2012; Krishna and Schwarz, 2013; Agapito et al., 2014; Barnes et al., 2014). These positive sensory experiences can create a strong bond and identification between the visitor and the destination. This strong bond and identification can influence visitors’ level of digital engagement while in the destination and after going back home (Lv et al., 2020b; Li et al., 2021). As such, visitors who have a strong bond and identification with the destination are more likely to share their positive experiences on social media platforms than those with a relatively low attachment.

Therefore, the purpose of this study is to explore whether sensory experiences in a destination can influence visitors’ digital engagement through the dependence on and identification with a destination. Although some previous studies have examined the impacts of travel motivations (Su et al., 2020) and smart tourism on travelers’ digital engagement level (Krisna et al., 2019), the antecedents of visitors’ digital engagement still largely remain underexplored in the tourism literature. This study investigates the influence of destination sensory experiences on visitors’ digital engagement with tourism destinations and sheds light on the underlying mechanism. In addition, this study helps the destination to gain competitive advantage in the fierce competition.

## Literature Review and Conceptual Framework

### Visitors’ Digital Engagement

Visitor engagement refers to real-time interactions among tourists, local communities, and destinations (Brodie et al., 2011). The importance of visitor engagement in enhancing satisfaction and loyalty has been recognized by destination marketers and managers (Chen and Rahman, 2018; Su et al., 2020). Engaged visitors are also reported to be active advocates of a destination in online and offline settings (Goh et al., 2013). Furthermore, contents generated and shared online by engaged visitors tend to be much more detailed and convincing (Goh et al., 2013; Park et al., 2018) than those shared by others. Thus, destination marketers and managers have developed various marketing and promotion programs (e.g., the reward system) to promote visitor engagement in online and offline settings (Ahn and Back, 2018). While visitors could engage with a destination in a variety of ways (e.g., positive WOM behavior, commenting, reviewing, and sharing selfies or videos to spread contents related to the destination), social media (Ni et al., 2020; Liu et al., 2021) and third-party review sites have been the dominant enablers of visitors’ digital engagement with a destination.

Visitor digital engagement refers to a visitor’s online interactions with a destination, other potential visitors, and his/her own social networks (e.g., friends, colleagues or followers) through various digital channels. These channels include third-party review sites, microblogging sites, social media, and many more. Given that visitors can create and exchange destination’s information and share their experiences with the destination easily on social media, visitors’ digital engagement with destinations and other tourism products and services through social media has been investigated in many studies (Cabiddu et al., 2014; Cheng and Edwards, 2015; Hudson et al., 2015).

Most previous studies on visitor engagement have focused on the consequence of engagement and found that visitor engagement can generate positive outcomes (e.g., a higher level of satisfaction, loyalty, and emotional commitment, positive WOM) for destinations and tourism providers. However, the antecedents of visitor engagement in the context of tourism only received limited attention. Studies that investigated the antecedents of engagement have identified involvement (Hollebeek et al., 2014; Gao and Lan, 2020) and a destination’s relevance to a visitor’s needs, values, and interests (Zaichkowsky, 1994) as critical antecedents. Since visitors’ sensory experience can enhance visitors’ positive feelings toward and their cognitions of a destination, which can help visitors form positive emotional responses toward a destination, visitors’ sensory experience can further influence visitors’ psychological and behavioral connections to the destination. Thus, visitors’ sensory experience can influence their level of digital engagement with a destination.

### Visitors’ Sensory Experiences

The experience economy, coined by Pine and Gilmore (1998), suggests that delivering unforgettable experiences is critical for the success and survival of brands since unforgettable experiences can produce significantly more positive customer outcomes than just delivering goods and services (Pine and Gilmore, 1998). This is especially true for the tourism industry, as delivering unforgettable hedonic experiences is the core focus of the industry (Mossberg, 2007). Since the interactions between visitors and external environments are all experienced through sensory channels (Libet et al., 1979), visitors’ sensory experiences can have a significant impact on how visitors evaluate their overall destination experiences. While most studies that have addressed the importance of sensory experiences on visitors’ behaviors’ (Gretzel and Fesenmaier, 2010; Chen and Lin, 2018) have mainly investigated the effects of visual cues (Agapito et al., 2017), a small number of studies have also investigated the effects of non-visual senses such as auditory sense (Richards et al., 2010; Small et al., 2012), multiple senses (Pan and Ryan, 2009), or all five senses (Agapito et al., 2014; Agapito et al., 2017; Lv et al., 2020b; Lv and Wu, 2021) on visitors’ behaviors.

An individual’s travel experience involves establishing a connection with a destination or travel activity through all sensory channels (Pan and Ryan, 2009). These sensory experiences and connections yield physical sensations that determine how visitors evaluate their travel experiences (Agapito et al., 2017). Thus, visitors’ sensory experiences play critical roles during the perception, emotion, and behavioral intention formation processes (Krishna, 2012; Agapito et al., 2014; Barnes et al., 2014). By delivering experiences that targets visitors’ five senses, a destination can improve visitors’ satisfaction with their sensory experiences, which can enhance their experience’s qualities and value perceptions. These enhanced qualities and value perceptions can help establish a bond or strengthen an existing bond between a destination and a tourist. This bond can result in the formation of a physical attachment to (the dependence and identification with) a destination (Lv et al., 2020b).

Pleasant and memorable sensory experiences can help create highly engaged visitors through influencing visitors’ emotional attachment to a destination (Lv and Wu, 2021). For instance, the visual and auditory elements of tourism activities in a destination influence visitors’ sensory experiences. These sensory experiences can further motivate visitors to form a strong emotional attachment to the destination and promote visitors to engage in destination-related activities, including online activities such as playing destination-themed videogames (Eigenraam et al., 2018), participating in virtual reality and social media activities (Tussyadiah et al., 2018). Digital engagement is visitors’ continuous and frequent interactions with a destination through the internet and reflects the strength and continuity of the relationship between visitors and the destination. Visitors’ digital engagement with a destination may also be influenced by their sensory experiences.

Based on the preceding discussion, this study proposes the following hypothesis:

H1: *Sensory experiences have a positive effect on digital engagement.*

### The Mediating Role of Place Attachment

Satisfactory sensory experiences in a destination could strengthen the relationship between visitors and the destination, which can result in the formation of an emotional, psychological, and behavioral connection to the destination (Brodie et al., 2011; Dessart et al., 2015). In other words, satisfactory sensory experiences can guide visitors to form a special attachment to the destination.

Place attachment refers to a positive emotional connection between the place and the self (Gross and Brown, 2008). In the tourism field, place attachment reflects the level of emotional bond between a destination and visitors. Place attachment is usually considered to be a multi-dimensional construct that includes place dependence (Kyle et al., 2003; Gross and Brown, 2008; Yuksel et al., 2010; Tsai, 2012; Maricchiolo et al., 2021), place identity (Kyle et al., 2003; Yuksel et al., 2010; Tsai, 2012), affective attachment (Kyle et al., 2003; Yuksel et al., 2010; Ramkissoon et al., 2012; Tsai, 2012), and social bonding (Kyle et al., 2004; Ramkissoon et al., 2012; Kim et al., 2016). Considering the interdependency between these three dimensions, most previous studies conceptualized place attachment as having two sub-dimensions: place dependence and place identity (Lee and Shen, 2013; Woosnam et al., 2016).

Place dependence, also known as functional attachment, refers to the functional relationship between a person and a place that aims to satisfy the functional needs of a person or accomplish a specific functional goal (Stokols and Schumaker, 1981). Visitors acquire destination dependence because a destination can provide the physical environments and tourism facilities such as attractions, facilities, amenities, and activities visitors would like to experience (Hernández et al., 2007). Since the quality of those physical environments and tourism facilities can influence the quality of visitors’ sensory experiences, the creation of pleasant and unforgettable sensory experiences through interacting with those physical environments and tourism facilities in a specific destination can increase visitors’ dependence on that destination for satisfactory sensory experiences.

Based on the preceding discussion, this study proposes that:

H2: *Sensory experiences have a positive effect on place dependence.*

Place identity refers to special and symbolic meaning of a place for a person. Individuals who have emotional connections and bonds with a place are likely to identify themselves with that place due to their feeling that they belong to that specific place (Breakwell, 1986). Since the quality of sensory experiences in a destination can significantly improve the emotional relationship between visitors and a destination (Moreira et al., 2017), pleasant and unforgettable sensory experiences can result in the formation of emotional connection and special bond with a visitor and a destination (Phillips et al., 2011). This emotional connection and the special bond can lead a visitor to identify himself or herself with that destination due to the feeling that they belong to that destination (Lian Chan and Baum, 2007).

Based on the preceding discussion, this study proposes that:

H3: *Sensory experiences have a positive effect on place identity.*

Place attachment resulting from the strong attachment and connection to a destination will further improve visitors’ psychological, emotional, and behavioral connection to the destination (Kastenholz et al., 2018). The strength of this attachment can influence visitors’ involvement and engagement with the destination during and after their visit to the destination (Villamediana et al., 2019). Since most post-visit engagement takes place online, visitors’ level of attachment to a destination should influence visitors’ level of digital engagement with a destination (Manzo and Perkins, 2006). Furthermore, place attachment may play a mediating role in the effect of visitors’ sensory experiences on their digital engagement with a destination.

Based on the preceding discussion, this study proposes that:

H4: *Place dependence has a positive effect on digital engagement.*
H5: *Place identity has a positive effect on digital engagement.*

**FIGURE 1:**
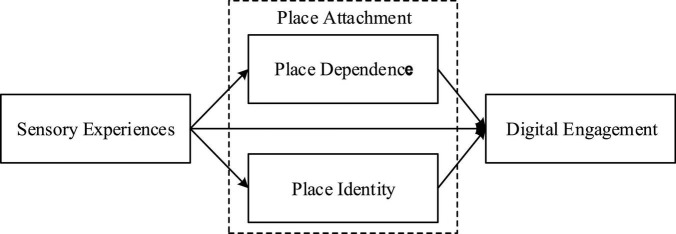
Conceptual framework.

Drawing from theories of customer engagement, sensory experiences, place attachment as well as previous tourism research, a conceptual framework that presents the proposed hypotheses is depicted in Figure 1. The conceptual model suggests that visitors’ sensory experiences influence both dimensions of place attachment, and those dimensions determine visitors’ level of destination attachment.

## Materials and Methods

Proposed hypotheses were tested utilizing data that were collected through a two-step process. First, visitors’ sensory experiences in a tourist destination were identified through a content analysis of online review comments posted by visitors. Afterward, a self-administered survey questionnaire was used to gather data from visitors who visited a specific destination to test the effects of those sensory experiences on visitors’ digital engagement through place dependence and place identification are examined.

### Measurement Items

Measurement items were developed utilizing a two-step process. First, visitors’ sensory experiences were identified by analyzing the online reviews posted by visitors about Sanya, a popular Chinese destination with tropical coastal scenery. Sanya is known as “Oriental Hawaii.” Sanya was chosen as the study site due its abundance of tourism sensory stimulation activities, which provide visitors with various sensory experiences, and numerous tourist online reviews. Online reviews of Sanya on Ctrip (the largest Chinese online travel platform) were extracted and used to determine the sensory experiences which visitors have in Sanya. Online reviews were chosen over questionnaires since they can provide more objective information about visitors’ destination experiences without being influenced by researchers (Zaichkowsky, 1994; Lv et al., 2020a).

A content analysis method was utilized to code the sensory experiences. According to Agapito et al. (2017) and Lv et al. (2020b), sensory impressions (visitors’ memories of sensory experiences) are usually used to measure sensory experiences. The first 2000 high-quality reviews with more than 50 words were selected, and after excluding repeated and obvious advertising reviews, 1896 valid online reviews were collected. Three trained doctoral students coded visitors’ sensory experiences separately. The coding process continued until no new sensory experience could be identified. Through this process, 33 sensory experience items were derived from online reviews. The coding consistency coefficient among coders was 0.853, indicating a sufficient reliability. Item frequencies are presented in Appendix 1.

According to previous studies, visitors’ sensory experiences could be both positive and negative, and cannot be simply added up (Lv et al., 2020a,b). However, coding results of online reviews about Sanya suggested that visitors mainly had positive sensory experiences in Sanya. Therefore, only positive sensory experiences were measured in this survey.

Afterward, a survey questionnaire was developed based on coding results and items identified from previous studies (e.g., Agapito et al., 2017). Visitors’ sensory experiences were measured using items identified from online reviews (see Appendix 1) utilizing a seven-point scale (1 = Not impressive at all, 7 = Very impressive). Place dependence, place identity (Williams and Vaske, 2003), and digital engagement (Schivinski et al., 2016) were each measured by six items, with a 7-point Likert scale (1 = strongly disagree, 7 = strongly agree; see Table 1).

**TABLE 1:** Measurement items.

Items	Source
**Sensory experiences**	Lv et al., 2020a
How impressed were you with the following sensory stimuli of Sanya during your visit? (33 items in Appendix 1)	
**Place dependence**	Williams and Vaske, 2003
1. Sanya is the best place for what I like to do	
2. No other place can compare to Sanya	
3. I get more satisfaction out of visiting Sanya than any other	
4. Doing what I do at Sanya is more important to me than doing it in any other place	
5. I wouldn’t substitute any other area for doing the types of things I do at Sanya	
6. The things I do at Sanya I would enjoy doing just as much at a similar site	
**Place identity**	Williams and Vaske, 2003
1. I feel Sanya is a part of me	
2. Sanya is very special to me	
3. I identify strongly with Sanya
4. I am very attached to Sanya	
5. Visiting Sanya says a lot about who I am	
6. Sanya means a lot to me	
**Digital engagement**	Schivinski et al., 2016
1. I post comments about my experiences, photos and videos on social media such as WeChat and Weibo	
2. I repost my experiences, photos and videos on social media such as WeChat and Weibo	
3. I frequently browse information, pictures, etc., related to the destination on social media	
4. I “Like” posts related to destination Sanya	
5. I read other people’s comments and commented on posts related to the destination and	
6. I follow the destination-related accounts on social media	

### Sample and Data Collection

Data for this study were collected in Sanya (a famous seaside destination in China) from September to November 2019. A total of 396 responses (350 valid) were collected, with an effective rate of 88.4%. As presented in Table 2, 43.71% of the respondents were male, and 56.29% of them were female. More than one-third of participants were between 18 and 25 years old (38.57%), with a monthly income under 5000 CNY (42.86%). Most participants (76.29%) had a college degree or above.

**TABLE 2:** Demographic characteristics.

Characteristics	Number	Percentage (%)
**Gender**		
Male	153	43.71
Female	197	56.29
**Age**		
16–18	7	2.00
18–25	135	38.57
26–35	95	27.14
36–45	41	11.71
46–60	44	12.57
Over 60	28	8.00
**Education level**		
High school or below	35	10.00
College/University	267	76.29
Master or doctoral	48	13.71
**Income (Monthly, CNY)**		
<1001 CNY	11	3.14
1001–5000 CNY	139	39.71
5001 CNY–8000 CNY	129	36.86
8001 CNY–17,000 CNY	55	15.71
17,001 CNY–30,000 CNY	12	3.43
>30,001 CNY	4	1.14

## Data Analysis and Results

### Measurement Model

Cronbach’s tests revealed high internal reliability of the items that measured place dependence (α = 0.920), place identity (α = 0.935) and digital engagement (α = 0.928). The measurement model was assessed through the confirmatory factor analysis (CFA). The fit indexes revealed that the measurement model had an adequate fit: χ^2^ = 176.231, df = 132, χ^2^/df = 1.34 < 2, RMSEA = 0.031 < 0.08, GFI = 0.949 > 0.9, CFI = 0.991 > 0.9, NFI = 0.966 > 0.9. The values of composite reliability (CR) were in the range of 0.921–0.935, exceeding the recommended threshold of 0.70, which indicated a high reliability. The magnitudes of standardized factor loadings for all items were between 0.801 and 0.872, all values were statistically significant, indicating that the indicators adequately represent the reflective factors (see Table 3). The values of average variance extracted (AVE) score for all constructs were in the 0.660–0.707 range, greater than 0.50 (Fornell and Larcker, 1981), indicating a high convergent validity (see Table 4). The inter-correlations of constructs were less than the square root of AVE for each construct, suggesting good discriminating validity (Fornell and Larcker, 1981).

**TABLE 3:** Results of confirmatory factor analysis.

	Mean	SD (*n* = 350)	Cronbach’ α	Factor loading (CFA)	AVE	Composite reliability
Place dependence	5.80	0.682	0.92		0.660	0.921
PD1	5.81	0.792		0.801		
PD2	5.79	0.849		0.810		
PD3	5.76	0.756		0.818		
PD4	5.87	0.857		0.821		
PD5	5.78	0.804		0.814		
PD6	5.75	0.779		0.809		
Place identity	5.50	0.771	0.935		0.707	0.935
PI1	5.49	0.899		0.872		
PI2	5.49	0.863		0.818		
PI3	5.49	0.879		0.843		
PI4	5.46	0.868		0.818		
PI5	5.52	0.911		0.828		
PI6	5.53	0.901		0.865		
Digital engagement	5.69	0.707	0.928		0.683	0.928
DE1	5.74	0.774		0.850		
DE2	5.66	0.823		0.812		
DE3	5.70	0.835		0.828		
DE4	5.70	0.841		0.810		
DE5	5.69	0.813		0.830		
DE6	5.64	0.864		0.826		

**TABLE 4:** Means, SD, Cronbach’α, CR and AVE.

Variables	Mean	SD	Cronbach’ α	AVE	CR	1	2	3	4
1. Sensory experiences	5.43	0.804							
2. Place dependence	5.80	0.682	0.920	0.660	0.921	0.676	[0.812]		
3. Place identity	5.50	0.771	0.935	0.707	0.935	0.613	0.415	[0.841]	
4. Digital engagement	5.69	0.707	0.928	0.683	0.928	0.575	0.511	0.704	[0.826]

*n = 350; CR, composite reliability; AVE, average variance extracted; All correlations are significant at the 0.01 level; [] is the square root of AVE.*

To control the common method bias (Podsakoff et al., 2003), the procedural remedies suggested by Tehseen et al. (2017) were used in the design and distribution of survey questionnaires. Furthermore, the Harman’s single-factor test was utilized to examine common method bias. The first component with the largest eigenvalue explained 39.2% variance (below 50%), which suggested that there was no common method bias in the collected data (Luo et al., 2016).

### Structural Model

Structural equation modeling (SEM) utilizing the maximum likelihood estimation method was employed to examine our hypotheses. The overall fit of the structural model was as follows: χ^2^ = 319.505, df = 148, χ^2^/df = 2.159 < 3, RMSEA = 0.058 < 0.08, GFI = 0.919 > 0.9, CFI = 0.967 > 0.9, NFI = 0.941 > 0.90. These results provided evidence of a good model fit (Hair et al., 2010).

Path analysis was conducted to examine the proposed hypotheses. The structural model results are shown in Table 5. The effect of sensory experiences on digital engagement was first tested. The standardized path coefficient between sensory experiences and digital engagement was 0.531 (t = 11.684), indicating that the effect of sensory experiences on digital engagement was significant, which provided support for H1.

**TABLE 5:** Pairwise parameter comparisons.

Hypothesis	Path	β	*t*-value	Results
H1	Sensory experiences → Digital engagement	0.531	11.684***	Supported
H2	Sensory experiences → Place dependence	0.676	13.573***	Supported
H3	Sensory experiences → Place identity	0.613	12.809***	Supported
H4	Place dependence → Digital engagement	0.226	3.264***	Supported
H5	Place identity → Digital engagement	0.564	8.471***	Supported

***p < 0.001.

The standardized path coefficient between sensory experiences and place dependence was 0.676 (t = 13.573), suggesting that sensory experiences significantly affects place dependence. Therefore, H2 was supported. The standardized path coefficient between sensory experiences and place identity was 0.613 (t = 12.809), indicating that place identity is effectively influenced by sensory experiences. Therefore, H3 was supported.

The direct effect of place dependence on digital engagement (β = 0.226, t = 3.264) was significant. Similarly, the direct effect of place identity on digital engagement (β = 0.564, t = 8.471) was significant. These findings provided support for H4 and H5. The direct effects, indirect effects and total effects are presented in Table 6.

**TABLE 6:** Direct effect, indirect effect, and total effects.

	Effect	Sensory experiences	Place dependence	Place identity
Place	Direct effect	0.676		
dependence	Indirect effect	–		
	Total effects	0.676		
Place	Direct effect	0.613		
identity	Indirect effect	–		
	Total effects	0.613		
Digital	Direct effect	–	0.226	0.564
engagement	Indirect effect	0.499	–	–
	Total effects	0.575	0.226	0.564

## Conclusion and Discussion

### Conclusion

The primary goal of this study was to explore how visitors’ sensory experiences affect their digital engagement with a destination and the mechanism of this effect. First, a content analysis was conducted on review comments shared by travelers who visited Sanya to identify their sensory experiences. Results of the content analysis showed that visitors could spontaneously have rich positive sensory experiences in a destination and those sensory experiences could improve visitors’ place attachment to the destination. Afterward, a SEM analysis was conducted. Results revealed that sensory experiences positively affect digital engagement with a destination, and this effect is mediated by place dependence and place identity.

Sensory experiences effectively facilitate and stimulate visitors’ digital engagement with a destination. These findings suggest that senses are the most direct way for people to feel the world around themselves, and compared to a perceived abstract destination image, sensory experience is much powerful in stimulating visitors’ destination loyalty and re-visit intentions (Libet et al., 1979; Lv et al., 2020b). The results clearly suggest that each positive sensory experience in a destination contributes to the generation of the overall memorable sensory experiences in a destination, which leads to positive feelings, satisfaction and ultimately contributes to positive attitudes and behaviors toward that destination. These findings are consistent with findings reported in previous studies that tourists’ sensory experiences are a better and more complete reflection of their overall experiences with a destination (Gentile et al., 2007; Brakus et al., 2009).

Findings also suggest that intense and unforgettable sensory experiences can lead to visitors’ place dependence. Those sensory experiences allow visitors to develop positive feelings toward a destination during their visits, in which result in the formation of close personal relationships with and a strong attachment to the destination. Those close personal relationships and the strong attachment also help visitors to form an emotional identification with the destination. Visitors who develop a strong identification with a destination recognize that destination as a part of who they are. These close personal relationships between visitors and destinations and the resulting dependence and identification increase visitors’ online engagement with the destination. In the age of social media, these highly attached visitors who depend on and identify themselves with the destination are more willing to share their experiences and feelings about the destination through written comments, photos and videos on social media sites like Twitter, Instagram and Facebook, which clearly suggesting a high digital engagement with the destination.

### Theoretical Implications

Although customer engagement has received significant research attention, this study revisits this important topic from the perspective of sensory marketing and embodied cognition perspective by highlighting the influence of sensory experiences in a destination on visitors’ digital engagement with that destination. Previous studies have explored how to encourage visitors to share their positive experiences with others, such as posting comments, pictures, videos, etc., to review sites and sharing those through various social media sites (Litvin et al., 2008), which are generally motivated by visitors’ level of satisfaction or dissatisfaction with their experiences (Su et al., 2020), rewards offered by destinations for sharing positive experiences (Dessart et al., 2015), and personal and cultural factors (Zhang et al., 2019). In contrast, this study examines how sensory experiences in a destination can affect visitors’ digital engagement with the destination. The findings of this study enhance our understanding of the antecedents of visitors’ digital engagement with a destination by employing a “sensory – behavioral” approach.

Findings also suggest that sensory experiences are critical antecedents of visitors’ bond and identification with a destination. Visitors’ positive destination-related sensory experiences increase their dependence on and identification with the destination, and this attachment and identification positively influence their digital engagement behavior on social media. Thus, this study sheds light on the mechanism of how sensory experiences influence visitors’ digital engagement with a destination through establishing a strong bond and identification between the destination and visitors. These findings provide empirical evidence that place attachment and identification are key emotional behaviors that can facilitate visitors’ digital engagement with a destination.

### Managerial Implications

In the era of Web 3.0, user-generated content shared on various social media channels profoundly impacts our lives. The popularity of online bookings and social media has radically changed the way visitors interact with destinations and make their purchasing decisions (Yin et al., 2017). In fact, experiences and comments shared about destinations, various products and services offered in a destination have become important information sources for consumers that can have significant impacts on the decision-making process (Godnov and Redek, 2016). Furthermore, information shared by previous visitors of a destination has been considered to be more reliable by potential visitors than the information received through traditional marketing channels (Gunter and Oender, 2016). Thus, how to motivate visitors to share their experiences online through posting positive reviews, comments, pictures and videos, and to further motivate those visitors to respond to those posts have become an important concern for destination managers and marketers. Thus, visitors’ digital engagement with a destination has become an important topic for both researchers and practitioners alike.

Visitors’ digital engagement with a destination before, during and after a visit is critical for a destination’s development, prosperity, and survival. Thus, it is important for destination managers and marketers to understand the factors that can increase visitors’ digital engagement with a destination. As sensory experiences in a destination can enhance visitors’ digital engagement behavior, destinations should actively create various unforgettable and unique sensory stimulus to help visitors form an overall positive sensory experience perception. Specifically, destination managers and marketers need to identify the sensory experiences that are most valued by visitors through marketing research and develop experiences that can satisfy those sensory needs and wants. Destinations can easily identify those sensory expectations through analyzing online review comments and responses to those comments. They can also use the expectations of sensory experiences identified through the content analysis of online reviews as a segmentation tool to identify groups of customers who value similar sensory experiences. For example, the sensory taste experience of most Sanya visitors in this study comes from their experiences with seafood and local food. Therefore, it makes more sense for the destination managers and marketers of Sanya to emphasize the availability of various seafood and local culinary options in their marketing communications, in order to address visitors’ needs and wants for sensory taste experiences. As argued by Agapito et al. (2014), various groups of visitors are likely to pursue different sensory experiences. Thus, using expectations of sensory experiences might be a good way of segmenting the market.

Previous studies have mostly focused on visual sensory experiences even though visitors experience a destination through various sensory channels such as visual, auditory (Jiang et al., 2017; He et al., 2018), olfaction (Dann and Jacobsen, 2003), taste (Everett, 2008) and tactile (Lv et al., 2020a). Thus, identifying visitors’ expectations regarding all five sensory channels can enable destination managers to develop products and services that can target each sensory expectation and deliver rich sensory experiences visitors expect (or hope) to have. Destination managers and marketers are strongly urged to monitor the sensory experience’s expectations of each target market and change in those sensory experience’s expectations in order to modify/update the product and service offerings to meet or exceed visitors’ sensory experience expectations. This approach can ensure visitors’ digital engagement with the destination while they are in the destination and after they return home, which can help the destination to generate a competitive advantage over other destinations in the fiercely competitive marketplace.

## Research Limitation

While the findings of this study provide critical insights to both researchers and practitioners by investigating the relationship between sensory experiences and digital engagement, this study is not free from limitations. This study only focuses on positive sensory experiences. Since negative sensory experiences can also have a significant impact on visitors’ attitudes and behaviors toward a destination, future studies should investigate the effects of both positive and negative sensory experiences on the digital engagement with a destination. Furthermore, this study solely considered the influence of the overall sensory experiences on visitors’ digital engagement with a destination. Future research could investigate how five sensory experiences influence the overall sensory experiences on visitors’ digital engagement, respectively. Since sensory experiences through different sensory channels might have varying influences on the perceptions of the overall sensory experience, the effects of sensory experiences experienced through different sensory channels on engagement levels could be examined. Another limitation of this study is that this study only investigated the sensory experiences of visitors to a single destination. Future research should investigate visitors’ sensory experiences in a variety of destinations to expand the findings’ external validity. Data for this study were collected through a survey methodology to test the hypotheses. To enhance the internal validity, future research could apply experimental methods to examine the cause-and-effect relationships between the variables.

## Data Availability Statement

The original contributions presented in this study are included in the article/supplementary materials, further inquiries can be directed to the corresponding author.

## Author Contributions

JA: conceptualization, made further reviewing and editing the manuscript. LY: writing – original draft. YH: theoretical building of the manuscript and editing. YL: methodology, data curation, and collection and analysis the data. All authors discussed the structure of the manuscript and finalized the manuscript.

## Conflict of Interest

The authors declare that the research was conducted in the absence of any commercial or financial relationships that could be construed as a potential conflict of interest.

## Publisher’s Note

All claims expressed in this article are solely those of the authors and do not necessarily represent those of their affiliated organizations, or those of the publisher, the editors and the reviewers. Any product that may be evaluated in this article, or claim that may be made by its manufacturer, is not guaranteed or endorsed by the publisher.

## Appendix

**APPENDIX 1:** Coding results.

Sensory impression	Content	% of coding
**Visual**		
SI1 Waterscape	Sea, seawater	9.33
SI2 Resort	Room layout, scenery, recreational facilities	8.41
SI3 Tropical plants	Coconut palm, etc.	6.69
SI4 Sky/Sun	Sky, cloud, sun	6.67
SI5 Beach	White, soft sand	4.59
SI6 Internet-famous site	Scenic spots entrance, Shooting scenes	4.08
SI7 People	Citizens, vendors, tourists	2.66
SI8 Religious facility	Temple, GuanYin Buddha statue	2.66
SI9 Infinity pool	Infinity pool	2.47
SI10 Tropic flowers	Tropical flowers	2.33
SI11 Undersea	Tropical fish, coral, jellyfish, etc.	2.00
SI12 Shows	Folk customs performance	1.94
SI13 Seashore rocks	Rocks	1.94
SI14 Ship	Sailing boat, yacht, etc.	1.54
SI15 Shell/crab	Shells, crabs, white corals, etc.	1.26
SI16 Night scene	Night view of island and city	1.18
SI17 Animals	Birds, peacocks, alpacas, cats, dogs, etc.	1.08
SI18 Street view	Urban road and street	1.03
**Auditory**		
SI19 Wave	Sea washes the shore	0.64
SI20 Quiet environment	Silent	0.61
SI21 Music	Stores, bars, etc.	0.45
**Gustatory**		
SI22 Fresh air	Rich-oxygen air	0.93
SI23 Sea breeze	Sea breeze	0.18
**Olfactory**		
SI24 Seafood	Fresh seafood	7.05
SI25 Local food	Cantonese cuisine, wenchang chicken, etc.	4.53
SI26 Local snack	Baoluo noodle, qingbuliang, etc.	2.08
SI27 Tropical fruits	Mango, coconut, jackfruit, etc.	2.02
SI28 Local beverage	Local beer, fresh juice, etc.	1.50
**Haptic**		
SI29 Seawater	Playing by the sea, swimming, etc.	3.47
SI30 Sun tanning	Bright sunshine	2.23
SI31 Soft sand	Soft fine sand, play with sand	2.18
SI32 Comfortable temperature	Comfortable temperature	1.30
SI33 Wind	The feeling of sea breeze	1.21

*Because there are too many coding items for visual, taste and haptic, we choose to present items greater than 1%, while hearing and smell accounts for a relatively low proportion, thus, we keep all the items (Lv et al., 2020a).*
